# Relationship between oral status, swallowing function, and nutritional risk in older people with and without Parkinson's disease

**DOI:** 10.1590/2317-1782/20242023311en

**Published:** 2024-08-05

**Authors:** Ramon Cipriano Pacheco de Araújo, Cynthia Meira de Almeida Godoy, Lidiane Maria de Brito Macedo Ferreira, Juliana Fernandes Godoy, Hipólito Magalhães

**Affiliations:** 1 Programa Associado de Pós-graduação em Fonoaudiologia, Universidade Federal do Rio Grande do Norte – UFRN - Natal (RN), Brasil.; 2 Hospital Universitário Onofre Lopes – HUOL - Natal (RN), Brasil.; 3 Departamento de Fonoaudiologia, Universidade Federal do Rio Grande do Norte – UFRN - Natal (RN), Brasil.

**Keywords:** Parkinson Disease, Deglutition Disorders, Oral Health, Respiratory Aspiration, Malnutrition

## Abstract

**Purpose:**

To compare oral status, swallowing function (through instrumental and SLH assessment), and nutritional risk between dysphagic individuals with and without Parkinson's disease.

**Method:**

This is a cross-sectional retrospective study based on data collected from medical records. It included 54 dysphagic older adults, divided into two groups according to the diagnosis of Parkinson's disease. The study collected data on the speech-language-hearing assessment of postural control, tongue mobility and strength, maximum phonation time (MPT), and cough efficiency. Oral status was assessed using the number of teeth and the Eichner Index. The level of oral intake and pharyngeal signs of dysphagia were analyzed with four food consistencies, according to the International Dysphagia Diet Standardization Initiative classification, using fiberoptic endoscopic evaluation of swallowing, for comparison between groups. The severity of pharyngeal residues was analyzed and classified with the Yale Pharyngeal Residue Severity Rating Scale, and the nutritional risk was screened with the Malnutrition Screening Tool.

**Results:**

The group of older adults with Parkinson's disease was significantly different from the other group in that they had fewer teeth, unstable postural control, reduced tongue strength, reduced MPT, weak spontaneous coughing, pharyngeal signs, less oral intake, and nutritional risk.

**Conclusion:**

Dysphagic older people with Parkinson's disease had different oral status, swallowing function, and nutritional risk from those without the diagnosis.

## INTRODUCTION

The worldwide increase in life expectancy has increased the older population’s need for health services^(1)^. As they age, people undergo physiological changes in normal swallowing mechanisms that predispose them to swallowing disorders^(2)^. Hence, oropharyngeal dysphagia in older adults raises concern due to the need to manage their swallowing function efficiently and safely^(3)^. Such disorders can be worsened by aging-related comorbidities, such as Parkinson's disease (PD)^(4)^.

PD is the most common progressive neurodegenerative disorder, with an increasing prevalence in the population as they grow older^(5)^. This debilitating condition knowingly affects the central and peripheral nervous system, with a characteristic histopathological presence of beta-synuclein aggregates, popularly known as Lewy bodies and Lewy neurites^(6)^. Even though it involves degeneration of the nigrostriatal dopaminergic pathway, PD also impacts other neural pathways, which causes neuromediator dysfunctions, resulting in complex functional deficits evidenced mainly in hypophonia, dysarthria, dysphagia, and sialorrhea^(7)^.

The prevalence of dysphagia in PD is 36.9% worldwide and 28.9% in the American continent^(7)^. Despite the increasing early diagnosis in adults, dysphagia manifestations in PD are associated with the stage of the disease, the time of motor symptoms onset, and the presence of dementia symptoms^(7)^. Thus, most PD patients with severe dysphagia are at an advanced stage of the disease, such as IV and V on the Hoehn and Yahr scale^(8)^, with significant weight loss, sialorrhea, and dementia^(9)^. Although there is drug treatment for extrapyramidal signs, mainly to ease motor symptoms characteristic of the disease, increasing doses does not guarantee that dysphagia symptoms will improve, due to resistance to dopaminergic stimulation^(10)^.

Some common features of oropharyngeal dysphagia in PD include tongue tremor, mandibular bradykinesia, pharyngeal residue, somatosensory deficits^(11)^, and high frequency of silent aspirations, which can result in hospitalization for aspiration pneumonia in more severe cases^(5)^. Nevertheless, dysphagia is directly associated with malnutrition and aspiration pneumonia in PD, which is the main cause of death among patients with this diagnosis. Due to the reduced sensitivity and efficiency of the cough reflex, there is a greater chance of the individual aspirating fluids and saliva frequently, so such signs are neglected in the home environment by family members and closest caregivers^(5)^.

Thus, instrumental and speech-language-hearing (SLH) swallowing assessment is the main way of understanding the pathophysiology in this population and predicting possible changes that decline swallowing efficiency and safety. Dysphagic older individuals with PD are expected to have greater impairments in swallowing function and oral and nutritional status than dysphagic older individuals without the diagnosis. Therefore, this study aimed to compare oral status, swallowing function (through instrumental and SLH assessment), and nutritional risk between dysphagic individuals with and without PD.

## METHODS

This is a cross-sectional retrospective study based on data collected from medical records. The research was conducted in the otorhinolaryngology outpatient clinic of Hospital Universitário Onofre Lopes, where data was collected regarding the fiberoptic endoscopic evaluation of swallowing (FEES) of patients treated between 2017 and 2023. All participants or their legal guardians signed an informed consent form provided by the service before carrying out the exam procedures. The study was approved by the Research Ethics Committee of the Hospital Universitário Onofre Lopes, under evaluation report no. 6.169.294. The data were collected from a previous SLH exam, the FEES findings, and post-exam nutritional screening.

### Sample

The sample had 54 older adults chosen by convenience among individuals who sought care at the said location and stratified into two groups, according to whether they had a PD diagnosis. The first group (PDG) had 22 dysphagic individuals aged 60 to 86 years, with a mean of 71.3 (±8.5) years, a predominance of males (63.6%), and diagnosed with PD classified into stages I, II, and III, according to the Hoehn and Yahr scale^(8)^. The second group had 32 older people with clinical complaints of oropharyngeal dysphagia, aged 60 to 86 years, with a mean of 70.5 (± 7.8) years, a predominance of females (84.4%), and no history of PD. It was not possible to verify whether all participants in this group were under the influence of dopaminergic medications due to outpatient demand.

The exclusion criteria for both groups were patients unable to follow commands, with other neurological diagnoses, tracheostomy, a history of oncological treatment, orotracheal intubation, and hospitalization in the 12 months before the examination.

All individuals in the sample had clinical complaints of oropharyngeal dysphagia, tracked by other health professionals from the service and/or referred by other sectors of the hospital, without a standardized protocol for instrumental investigation of swallowing.

### Procedures

#### Clinical assessment

The subjects underwent SLH clinical assessment when they were admitted to the outpatient clinic before the instrumental examination. An SLH pathologist from the service with more than 10 years of experience in oropharyngeal dysphagia was responsible for carrying out the analyses. This assessment used the service's protocol, analyzing orofacial myofunctional aspects, oral status, phonation, and cough efficiency.

Oral status was assessed based on the number of remaining teeth, denture use, and distribution of occlusal support in the molar region, according to the Eichner Index^(12)^. The latter was determined by the vertical contact components existing between bilateral molars and categorized into three types: Class A, contact between four occlusal support zones; Class B, contact between one to three occlusal support zones; and Class C, without occlusal contact. The number of teeth was described with and without using dentures, while the Eichner Index was assessed using the usual occlusal support for chewing – i.e., their current prosthetic rehabilitation.

Tongue mobility and strength were subjectively assessed by the SLH pathologist, who asked the patient to protrude and lateralize the tongue and push it against a resisting gloved finger. The ability to correctly execute the desired commands and maintain isometric strength against finger resistance were adopted as normality criteria. Despite being qualitative measures that depend on the evaluator’s previous experience in comparing normality, tongue weakness was defined as brief muscle contraction and rapidly decreasing isometric movement when they used maximum voluntary tongue strength against the resisting gloved finger.

After giving an example, the evaluator asked the patient to emit the vowel "a" for as long as possible. The normality criteria were Maximum Phonation Time (MPT) of 14 s for women and 20 s for men. Then, they underwent an auditory-perceptual evaluation of the voice to verify roughness in the vowel emission. They were also required to cough strongly and spontaneously to evaluate the subjective efficiency in coughing when asked (efficient/weak) for eventual pharyngeal cleaning. All changes were described and noted to continue the instrumental swallowing assessment.

#### Instrumental swallowing assessment

FEES was carried out by a resident physician, accompanied by a responsible otorhinolaryngologist and an SLH pathologist with experience in oropharyngeal dysphagia, following the institution's protocol. They used a flexible fiberoptic rhinolaryngoscope manufactured by Olympus^®^, 3.2 mm in diameter, model LF-P, with an attached micro-camera and light source. The patient was instructed to remain seated throughout the examination; no topical anesthetic was used to introduce the instrument into the nasal cavity down to the hypopharynx. Pharyngeal sensitivity was checked by touching the epiglottis with the rhinolaryngoscope, causing a pharyngeal constriction. After the physician’s structural analysis, the SLH pathologist served food artificially flavored with diet juice powder, artificially colored with blue aniline, and thickened with an instant corn starch product. At the end, they were also served 8-g portions of crackers at will.

Food consistencies were evaluated according to the International Dysphagia Diet Standardization Initiative classification (IDDSI)^(13)^, offered in the following order: 2 (mildly thick liquid), 4 (extremely thick liquid), and 0 (thin liquid), served three times in a 5-mL metal spoon, while 7 (regular solid) was offered in a single serving.

The three previously mentioned professionals with experience in the examination interpreted and assessed them simultaneously, by consensus, and concluded whether they had multiple swallowing, posterior oral leakage, and pharyngeal residue in the valleculae and/or pyriform sinuses, according to the Yale Pharyngeal Residue Severity Rating Scale (YPRSRS)^(14)^ (1 – None; 2 – Pharyngeal Trace; 3 – Mild Residue; 4 – Moderate Residue; 5 – Severe Residue). The presence of laryngeal penetration and laryngotracheal aspiration was also evaluated. The following parameters were considered for analysis, starting from the first serving: multiple swallows, when there were more than two attempts to swallow the same serving^(15)^; posterior oral escape, due to premature food escape in the hypopharynx before triggering the swallowing reaction^(15)^; pharyngeal residue, by identifying colored food residue in the valleculae and/or pyriform sinuses after swallowing the first serving^(14)^; laryngeal penetration, via observation of colored food residue in the vocal folds^(16)^; and laryngotracheal aspiration, when there was colored food residue below the vocal folds^(16)^. All analyses were performed in real-time, and the images were stored on a computer at the outpatient clinic to be reviewed as many times as the professionals deemed necessary after carrying out the exam.

The level of oral intake was assessed by professionals after the examination, using the Functional Oral Intake Scale (FOIS)^(17)^ based on examination analysis and the existence and need for liquid thickening. FOIS scores range from 1 (no oral intake) to 7 (total oral intake without restrictions).

#### Nutritional risk

To assess nutritional risk, a nutritionist from the service applied the Malnutrition Screening Tool (MST)^(18)^, which has been translated and adapted into Portuguese with three questions on the self-perceived loss of weight and appetite for food in the last month. This is an accessible and quick instrument to apply to adults upon hospital admission; scores equal to or higher than two represent nutritional risk and the need for a more detailed nutritional assessment.

### Data analysis

Data were analyzed with descriptive and inferential statistics, using measures of central tendency, proportions, and frequencies. The Kolmogorov-Smirnov test was used to verify normal distribution in quantitative variables, followed by the Mann-Whitney test to compare the protocols and analyze the number of teeth before and after oral rehabilitation. For qualitative variables, such as “SLH assessment” and “videoendoscopic pharyngeal signs”, the Pearson Chi-square test or Fisher's Exact test was applied, depending on the frequency expected for each cell to be greater than or equal to 5. The significance level in all tests was set at 0.05.

## RESULTS

All participants in the group of older people without the diagnosis had dysphagia due to an idiopathic cause under investigation. Of the 22 PDG dysphagic individuals, 18.1% were in stage I, 54.5% in stage II, and 31.8% in stage III in the Hoehn and Yahr scale^(8)^. The relationship of SLH assessment findings between the groups is shown in Table 1, in which older adults with PD had unstable postural control, reduced tongue strength, reduced MPT, and weak spontaneous cough.

**Table 1:** Relationship between age, sex, and speech-language-hearing assessment findings between the groups

Variables	Groups		**p-value**
	With Parkinson	Without Parkinson	
	n = 22 (%)	n = 32 (%)	
Age (years)	71.32 (± 8.5)	70.50 (± 7.8)	
Sex			
Males	14 (63.6)	5 (15.6)	
Females	8 (36.4)	27 (84.4)	
**Speech-language-hearing assessment**			
Postural control (seated)			
Adequate	13 (59.1)	29 (90.6)	0.009**
Unstable	9 (40.9)	3 (9.4)	
Tongue mobility			
Adequate	16 (72.7)	27 (84.4)	0.324
Reduced	6 (27.3)	5 (15.6)	
Tongue strength			
Adequate	10 (45.5)	27 (84.4)	0.002*
Reduced	12 (54.5)	5 (15.6)	
Maximum phonation time			
Adequate	7 (31.8)	25 (78.1)	0.001^*^
Reduced	15 (68.2)	7 (21.9)	
Roughness			
Absent	12 (54.5)	20 (62.5)	0.559
Present	10 (45.5)	12 (37.5)	
Spontaneous coughing			
Efficient	16 (72.7)	30 (93.8)	0.041^**^
Weak	6 (27.3)	2 (6.3)	

All data are presented in numbers (%) or means (standard deviation)

* Pearson’s chi-square;

** Fisher’s exact test

**Caption:** n (%) = absolute and relative frequency

The oral status described in Table 2 showed that 54.5% of PD patients did not use any type of dentures, while in the group of older people without the diagnosis, 53.1% did not use it. PDG had fewer teeth before and after using dentures; also, the number of teeth tended to be higher in both groups after using dentures.

**Table 2:** Relationship of oral status between the groups

Oral status	Groups		p-value
	With Parkinson	Without Parkinson	
	n = 22 (%)	n = 32 (%)	
Number of teeth without dentures	13 (8-20.2)	18 (12-24)	0.047*
Number of teeth with dentures	20 (9.7-24.2)	24 (20.5-26)	0.015^*^

Eichner Index				
Class A	12 (54.5)	22 (68.8)	0.288	
Class B or C	10 (45.5)	10 (31.3)		
Salivary stasis				
Absent	21 (95.5)	32 (100)	0.407	
Present	1 (4.5)	0 (0.0)		

All data are presented in medians (25-75 interquartile range) or numbers (%)

* Mann-Whitney test

**Caption:** n (%) = Absolute and relative frequency

The FEES and nutritional risk findings, presented in Table 3, indicate decreased pharyngeal sensitivity to touch in 40.9% of participants with PD. There was a greater occurrence of pharyngeal residue, laryngeal penetration, and laryngotracheal aspiration with thin liquid (level 0) and pharyngeal residues and laryngeal penetration with mildly thick liquid (level 2). However, there was no difference in the pharyngeal signs of dysphagia with extremely thick liquid (level 4) and regular solid (level 7) between the groups. Moreover, PDG individuals were the only ones who had penetration and aspiration with all food consistencies used in this study.

**Table 3:** Relationship between pharyngeal signs in the fiberoptic endoscopic evaluation of swallowing, oral intake level, and nutritional risk between the risks

Pharyngeal signs per food consistency level (IDDSI)	Groups		p-value
	With Parkinson	Without Parkinson	
	n = 22 (%)	n = 32 (%)	
Pharyngeal sensitivity to touch			
Preserved	13 (59.1)	28 (87.5)	0.016*
Reduced	9 (40.9)	4 (12.5)	
Thin liquid (level 0)			
Multiple swallows			
Yes	1 (4.5)	1 (3.1)	0.653
No	21 (95.5)	31 (96.9)	
Posterior oral escape			
Yes	11 (50.0)	11 (34.4)	0.251
No	11 (50.0)	21 (65.6)	
Pharyngeal residues			
Yes	14 (63.3)	11 (34.4)	0.034*
No	8 (36.4)	21 (65.6)	
Laryngeal penetration			
Yes	7 (31.8)	0 (0.0)	0.001**
No	15 (68.2)	32 (100)	
Laryngotracheal aspiration			
Yes	5 (22.7)	0 (0.0)	0.008**
No	17 (77.3)	32 (100)	
Mildly thick liquid (level 2)			
Multiple swallows			
Yes	1 (4.5)	1 (3.1)	0.653
No	21 (95.5)	31 (96.9)	
Posterior oral escape			
Yes	12 (54.5)	13 (40.6)	0.313
No	10 (45.5)	19 (59.4)	
Pharyngeal residues			
Yes	17 (77.3)	12 (37.5)	0.004*
No	5 (22.7)	20 (62.5)	
Laryngeal penetration			
Yes	4 (18.2)	0 (0.0)	0.023^**^
No	18 (81.8)	32 (100)	
Laryngotracheal aspiration			
Yes	2 (9.1)	0 (0.0)	0.161
No	20 (90.9)	32 (100)	
Extremely thick liquid (level 4)			
Multiple swallows			
Yes	0 (0.0)	1 (3.1)	0.593
No	22 (100)	31 (96.9)	
Posterior oral escape			
Yes	14 (63.6)	10 (31.2)	0.019^*^
No	8 (36.4)	22 (68.8)	
Pharyngeal residues			
Yes	13 (59.1)	12 (37.5)	0.118
No	9 (40.9)	20 (62.5)	
Laryngeal penetration			
Yes	3 (13.6)	0 (0.0)	0.062
No	19 (86.4)	32 (100)	
Laryngotracheal aspiration			
Yes	2 (9.1)	0 (0.0)	0.161
No	20 (90.9)	32 (100)	
Regular solid (level 7)			
Multiple swallows			
Yes	1 (4.5)	1 (3.1)	0.653
No	21 (95.5)	31 (96.9)	
Posterior oral escape			
Yes	4 (18.2)	3 (9.4)	0.425
No	18 (81.8)	29 (90.6)	
Pharyngeal residues			
Yes	4 (18.2)	3 (9.4)	0.425
No	18 (81.8)	29 (90.6)	
Laryngeal penetration			
Yes	3 (13.6)	0 (0.0)	0.062
No	19 (86.4)	32 (100)	
Laryngotracheal aspiration			
Yes	2 (9.1)	0 (0.0)	0.161
No	20 (90.9)	32 (100)	
Severity of residues (YPRSRS)	2.7 (± 1.1)	1.4 (± 0.5)	<0.001***
FOIS	5.1 (± 1.2)	6.0 (± 0.8)	0.006***
MST	1.0 (± 1.1)	0.2 (± 0.6)	0.004^***^

All data are presented in numbers (%) or means (standard deviation)

* Chi-square test;

** Fisher’s exact test;

*** Mann-Whitney test

**Caption:** n (%) = absolute and relative frequency; YPRSRS = Yale Pharyngeal Residue Severity Rating Scale; FOIS = Functional Oral Intake Scale; MST = Malnutrition Screening Tool

The analysis of the severity of residues in the valleculae and/or pyriform sinuses with YPRSRS found a difference in the classification between the groups – PDG had from traces to mild residue (YPRSRS 2-3). It was also noted that after the instrumental examination, PDG had FOIS level 5 (Total oral diet with multiple consistencies but requiring special preparation or compensations) and was at nutritional risk (Table 3).

## DISCUSSION

The exact mechanism that triggers dysphagia in PD is still unclear. However, various studies demonstrate the occurrence of different neuromuscular changes associated with swallowing as the disease progresses, impairing central and peripheral swallowing regulation mechanisms^(17)^. Therefore, the present study aimed to compare oral status, swallowing function (through instrumental and SLH assessment), and nutritional risk between dysphagic older adults with and without a PD diagnosis.

In the SLH assessment, a significant difference was found in postural control instability, reduced tongue strength, reduced MPT, and inefficient cough production. These clinical findings corroborate the understanding of aspects involved in impaired swallowing function, in comparison with older people without any such impairment^(9)^.

Tongue strength was an important measure with a significant difference between the groups, making it a finding consistent with previous evidence in this population. Thus, reduced tongue strength has been associated with PD through subjective measures (such as the SLH assessment) and objective measures (such as isometric tongue pressure in kilopascals [Kpa]), which cannot be modified with dopaminergic drug treatment^(19,20)^. In addition to being a clinical predictor of swallowing efficiency used in current research, low pressure between the tongue and palate also contributes to difficulties in managing and transporting the food bolus in the oral cavity^(21)^. It is also understood that the maximum pressure of the tongue is significantly reduced in more advanced disease stages, culminating in a stage with increased clinical swallowing complaints in this population^(22)^.

The research approached MPT and cough efficiency because they are reliable clinical parameters and provide SLH with important information about glottal closure and the efficiency of the lower airway protection reflex. PDG presented a difference between MPT and cough efficiency, which suggests low glottal closure resistance and a weak cough reflex to eject possible materials aspirated into the larynx. These results demonstrate that PDG has less swallowing protection and safety than those without the diagnosis, with greater risks of eventual silent aspirations when ingesting fluids, due to the weak cough reflex and the decrease in the sensory mechanism in the pharynx evidenced in other studies^(22,23)^.

Most PD patients did not have a constriction reflex when touched with the fiberoptic rhinolaryngoscope during FEES. This difference in pharyngeal sensitivity also strengthens the perspective of a sensory decrease in the pharyngeal phase in the present study. As these are important aspects related to the safety of swallowing, as the patient needs sensory mechanisms to trigger the swallowing reflex and eject remaining residues, their reduction has a negative impact on the efficiency and coordination of the food bolus in the pharyngeal phase^(24)^. These data also contribute to a deeper understanding of how the afference and efference in the pharyngeal phase, determined by involuntary mechanisms, may be impaired in PD pathophysiology. Although it was believed that dysphagia in PD emerged exclusively from weakened muscles, with inefficient movements in the oral and oral preparatory phases, many studies are dedicated to describing the pharyngeal phase of swallowing – which is relatively preserved in the initial stages of dysphagia, though with a progressive abnormality in sensorimotor integration between the oral and pharyngeal phases relevant to the dysphagic condition of the disease^(25)^.

Regarding oral status, the number of teeth before and after oral rehabilitation was different between older people with and without the diagnosis. Although the oral status is little researched in large PD samples, there is evidence of a weakening as the disease progresses, with periodontal diseases and dental cavities, resulting in more fragile and absent teeth in the oral cavity^(26)^. With compromised oral health and impaired oral motor function, dysphagic PD patients have greater complications in oral intake with a decline in dysphagia^(27)^. Because they have fewer teeth and, consequently, fewer occlusal contacts, PD patients need more time to chew and process food and may have changes in caloric intake due to preferring food consistencies that are easier to ingest^(27)^.

Although the analysis of occlusal contact between molars was not different between the groups, this information indicates that dysphagic individuals with PD in this sample had missing teeth. However, these absences were compensated with oral rehabilitation at the time of the Eichner Index assessment – particularly in the molar region, responsible for providing occlusal support and crushing food. In this context, denture use helps to improve both the oral and oral preparatory phases of swallowing. Reports indicate that the absence of dentures in toothless older people changes the anatomical structure and functional movements in the oral cavity, resulting in abnormal food bolus transport^(28)^.

The pharyngeal signs of dysphagia were analyzed by examining the FEES in different food consistencies. There was a difference between the groups with thin liquid and mildly thick liquid, as PDG had pharyngeal residue, laryngeal penetration, and laryngotracheal aspiration. These results demonstrate that liquid consistencies (i.e., those that flow easier) pose significant risks for the safety of swallowing in PD, in contrast with the group of dysphagic older people who do not have penetration or aspiration^(23)^. Thus, the data suggest that extremely thick liquids may be an alternative to increase swallowing safety and help understand that older adults with PD, even in the initial and intermediate disease stages (I, II, and III), are at risk of aspirating liquids.

Most of those in PDG had traces or mild residues (2-3), corroborating other evidence that dysphagia in PD is intrinsically related to the presence of residue after swallowing, which could be explained by the reduced tongue posterior propulsion and delayed swallowing reflex^(24)^. Residue measurement in PD is still not a consensus among studies, as they use heterogeneous assessment methods and sample recruitment. However, it is a fact that changes in food consistency reduce their occurrence^(23,29)^.

In the present study, there was a difference in the level of oral intake and nutritional risk, which suggests that older adults with PD have restrictions in oral intake and significant weight loss. The latter, along with malnutrition, is directly related to negative energy balance – i.e., an intake lower than energy expenditure, which in the case of dysphagia, occurs due to modifications and restrictions in food choices due to difficulty in swallowing. Therefore, there is evidence that PD patients are at greater risk of malnutrition than older people with dysphagia, due to the progressive decline in swallowing efficiency and safety and the impaired oral status^(30)^. Although the sample consisted of older adults, malnutrition is considered a secondary and subsequent factor to dysphagic manifestations and can be constantly monitored as markers of dysphagia in this population^(30)^. The prevalence of malnutrition in PD is still heterogeneous, depending on whether anthropometric or biochemical measurements or screening instruments are used^(30)^.

The limitations of the study include the small sample size in both groups, the lack of data on denture type and location, and the lack of anthropometric and/or biochemical data in the nutritional assessment for comparison, as these measurements could increase the number of individuals at nutritional risk. It is also important to mention the unequal number of participants at different PD stages, the time since diagnosis, and other information about the medications used, such as investigating whether participants were under the influence of medications during the exam. On the other hand, its strengths include the results, which can provide relevant information about the difference in the SLH and instrumental swallowing assessment findings between dysphagic older people with and without the diagnosis, enabling new hypotheses for clinical research involving these groups.

## CONCLUSION

There was a difference in oral status between the groups regarding the number of teeth, as well as in the SLH assessment, with reduced tongue strength, reduced MPT, weak spontaneous coughing, and oral intake. There were also signs of pharyngeal residue, laryngeal penetration, and laryngotracheal aspiration with thin and mildly thick liquid. Most dysphagic older people with PD were at nutritional risk.
