# Supplementary material for: Relationship between oral status, swallowing function, and nutritional risk in older people with and without Parkinson's disease
Source: Codas. 2024 Aug 5;36(5):e20230311. doi: 10.1590/2317-1782/20242023311en (PMC11340878; doi:10.1590/2317-1782/20242023311en)
Supplement: Translation

# Translation: Relação entre estado oral, função de deglutição e risco nutricional entre idosos com e sem doença de Parkinson

**DOI:** 10.1590/2317-1782/20242023311pt

**Authors:** Ramon Cipriano Pacheco de Araújo, Cynthia Meira de Almeida Godoy, Lidiane Maria de Brito Macedo Ferreira, Juliana Fernandes Godoy, Hipólito Magalhães

**Affiliations:** 1 Programa Associado de Pós-graduação em Fonoaudiologia, Universidade Federal do Rio Grande do Norte – UFRN - Natal (RN), Brasil.; 2 Hospital Universitário Onofre Lopes – HUOL - Natal (RN), Brasil.; 3 Departamento de Fonoaudiologia, Universidade Federal do Rio Grande do Norte – UFRN - Natal (RN), Brasil.

**Keywords:** Doença de Parkinson, Transtornos de Deglutição, Saúde Bucal, Aspiração Respiratória, Desnutrição

## Abstract

**Objetivo:**

Comparar o estado oral, a função de deglutição por meio da avaliação instrumental, fonoaudiológica e do risco nutricional entre indivíduos disfágicos com e sem doença de Parkinson.

**Método:**

Trata-se de um estudo transversal e retrospectivo com base na coleta de dados dos prontuários. Foram incluídos 54 idosos disfágicos divididos em dois grupos, de acordo com a presença do diagnóstico de doença de Parkinson. Foram coletados dados com relação à avaliação fonoaudiológica de controle postural, mobilidade e força de língua, Tempo Máximo de Fonação (TMF) e eficiência da tosse. O estado oral foi avaliado por meio do número de dentes e o Índice de Eichner. Foram analisados o nível de ingestão oral e os sinais faríngeos de disfagia em quatro consistências alimentares, de acordo com a classificação International Dysphagia Diet Standardisation Initiative (IDDSI), por meio da videoendoscopia da deglutição, para comparação entre os grupos. Para análise e classificação da gravidade dos resíduos faríngeos, foi utilizado o Yale Pharyngeal Residue Severity Rating Scale (YPRSRS), enquanto que, para rastrear o risco nutricional foi utilizado o Malnutrition Screening Tool (MST).

**Resultados:**

O grupo de idosos com doença de Parkinson apresentou diferença significativa em menor número de dentes, controle postural instável, força de língua reduzida, TMF reduzido, tosse espontânea fraca, sinais faríngeos, nível de ingestão oral menor e em risco nutricional, em comparação ao outro grupo.

**Conclusão:**

Os idosos disfágicos com doença de Parkinson apresentaram diferenças no estado oral, na função de deglutição e no risco nutricional em comparação àqueles sem o diagnóstico.

## INTRODUÇÃO

Com o crescimento da expectativa de vida da população mundial, há um aumento da procura por serviços de saúde pela população mais velha^(1)^. À medida que um indivíduo envelhece, ocorrem alterações fisiológicas nos mecanismos normais da deglutição que predispõe os distúrbios da deglutição^(2)^. Nesse contexto, a disfagia orofaríngea nos idosos torna-se uma preocupação pela necessidade de gerenciar a função de deglutição de forma eficiente e segura^(3)^. Esses distúrbios podem ser agravados por comorbidades associadas ao envelhecimento como a doença de Parkinson^(4)^.

A doença de Parkinson (DP) é o distúrbio neurodegenerativo progressivo mais comum, com uma prevalência crescente na população ao avançar da idade^(5)^. Sabe-se que essa condição debilitante afeta o sistema nervoso central e periférico, com presença histopatológica característica de agregados de beta-sinucleína, conhecidos popularmente como corpos de Lewy e neuritos de Lewy^(6)^. Mesmo que envolva a degeneração da via dopaminérgica nigroestriatal, a DP também impacta outras vias neurais, o que causa disfunções neuromediadoras, de modo que resultam em déficits funcionais complexos evidenciados, principalmente, na hipofonia, disartria, disfagia e sialorreia^(7)^.

A prevalência da disfagia na DP é de 36,9% no mundo e de 28,9% no continente americano^(7)^. Embora haja aumento do diagnóstico precoce na população adulta, as manifestações da disfagia na DP estão associadas ao estágio da doença, ao tempo de início dos sintomas motores e da presença de sintomas demenciais^(7)^. Assim, a maioria dos indivíduos com DP que possuem disfagia grave apresentam estágio avançado da doença, como estágio IV e V na escala de Hoehn e Yahr^(8)^, com perda de peso relevante, sialorreia e demência^(9)^. Apesar de haver tratamento medicamentoso dos sinais extrapiramidais, priorizado para aliviar os sintomas motores característicos da doença, o aumento de suas doses não garantem a melhora dos sintomas de disfagia, em decorrência da resistência à estimulação dopaminérgica^(10)^.

Algumas características comuns na disfagia orofaríngea na DP incluem o tremor de língua, bradicinesia mandibular, resíduos faríngeos, déficits somatossensoriais^(11)^ e alta frequência de aspirações silenciosas, o que pode resultar na hospitalização por pneumonia aspirativa em casos mais graves^(5)^. Não obstante, a disfagia está diretamente associada à desnutrição e a pneumonia por aspiração na DP, que é a principal causa de morte entre os pacientes com esse diagnóstico. Devido a sensibilidade e a eficiência no reflexo de tosse estarem reduzidas, há maiores chances do indivíduo aspirar fluidos e saliva frequentemente, de modo que tais sinais são negligenciados em ambiente domiciliar por familiares e cuidadores mais próximos^(5)^.

Dessa maneira, a avaliação instrumental e fonoaudiológica da deglutição tornam-se a principal forma de compreender a fisiopatologia nessa população e predizer as eventuais mudanças que levam ao declínio da eficiência e segurança da deglutição. Assim, espera-se que os indivíduos idosos disfágicos com DP apresentem maiores comprometimentos no estado oral, função de deglutição e estado nutricional, em comparação à idosos disfágicos sem o diagnóstico. Portanto, o objetivo deste estudo foi o de comparar o estado oral, a função de deglutição por meio da avaliação instrumental, fonoaudiológica e de risco nutricional entre indivíduos disfágicos com e sem doença de Parkinson.

## MÉTODOS

Trata-se de um estudo transversal e retrospectivo com base na coleta de dados dos prontuários. A pesquisa foi conduzida no ambulatório de otorrinolaringologia do Hospital Universitário Onofre Lopes, em que foram coletados dados referentes ao exame da videoendoscopia da deglutição (VED) dos pacientes atendidos entre 2017 a 2023. Todos os participantes, ou responsáveis legais, assinaram o Termo de Consentimento Livre e Esclarecido (TCLE) disponibilizado pelo serviço antes da realização dos procedimentos do exame. O estudo foi aprovado pelo Comitê de Ética em Pesquisa do Hospital Universitário Onofre Lopes - CEP, sob parecer n° 6.169.294. Os dados coletados foram referentes ao exame fonoaudiológico prévio, os achados da VED e a triagem nutricional pós exame.

### Casuística

A amostra foi constituída por 54 idosos escolhidos por conveniência entre os indivíduos que buscaram atendimento no referido local, que foi estratificada em dois grupos, de acordo com a presença do diagnóstico de doença de Parkinson. O primeiro grupo (GDP) foi composto por 22 indivíduos disfágicos com idade entre 60 a 86 anos, média de 71,3 (±8,5) anos e com predomínio do gênero masculino (63,6%), que apresentavam diagnóstico de doença de Parkinson classificados nos estágios I, II e III, segundo a escala de Hoehn e Yahr^(8)^. O segundo grupo, foi composto por 32 idosos com queixas clínicas de disfagia orofaríngea, com idade entre 60 e 86 anos, média de 70,5 (± 7,8) anos e com predomínio do gênero feminino (84,4%), que não apresentavam histórico de doença de Parkinson. Não foi possível inferir se todos os participantes desse grupo estavam sob efeito dos medicamentos dopaminérgicos devido a demanda ambulatorial.

Foram adotados como critérios de exclusão para ambos os grupos, a incapacidade de seguir comandos, outros diagnósticos neurológicos, usuários de traqueostomia, com histórico de tratamento oncológico, intubação orotraqueal e de hospitalização nos últimos 12 meses, previamente ao exame.

Vale ressaltar que todos os indivíduos da amostra apresentavam queixas clínicas de disfagia orofaríngea, rastreados por outros profissionais de saúde do próprio serviço e/ou encaminhados por outros setores do próprio hospital, sem um protocolo padronizado, para a investigação instrumental da deglutição.

### Procedimentos

#### Avaliação clínica

A avaliação clínica fonoaudiológica foi realizada previamente ao exame instrumental, no momento em que o paciente foi admitido no ambulatório. Um fonoaudiólogo do serviço com mais de dez anos de experiência em disfagia orofaríngea foi responsável por realizar as análises. Nessa avaliação foi utilizado o protocolo próprio do serviço, em que foram analisados aspectos miofuncionais orofaciais, estado oral, fonação e eficiência na tosse.

O estado oral foi avaliado pelo número de dentes remanescentes, uso de prótese e distribuição do suporte oclusal na região de molares, de acordo com o Índice de Eichner (IE)^(12)^. O IE foi determinado pelos componentes de contato vertical existentes entre os molares bilaterais e categorizados em três tipos: Classe A, contato entre quatro zonas de suporte oclusal, Classe B, contato entre uma a três zonas de suporte oclusal e Classe C, sem contato oclusal. O número de dentes foi descrito com e sem uso da prótese dentária, enquanto o IE foi avaliado utilizando o suporte oclusal habitual para mastigação, ou seja, com a utilização da reabilitação protética existente.

A mobilidade e força de língua foram aspectos subjetivos avaliados pelo fonoaudiólogo, que solicitou que o paciente executasse os movimentos desejados de protrusão e lateralização com a língua, e a protrusão de língua contra a resistência do dedo enluvado. Foram adotados como critérios de normalidade a capacidade de executar corretamente os comandos desejados e manter a força isométrica sobre a resistência do dedo. A fraqueza lingual foi observada quando o avaliador solicitava que o paciente utilizasse a força máxima voluntária de língua contra a resistência do dedo enluvado do próprio avaliador, que apesar de serem medidas qualitativas, a depender da experiência prévia de comparação da normalidade, a fraqueza resulta em uma baixa duração de contração muscular e diminuição rápida do movimento isométrico.

O avaliador ainda foi o responsável por solicitar ao paciente que emitisse o som da vogal "a" pelo máximo de tempo possível após modelo, adotando-se como critérios de normalidade o tempo cronometrado de 14s para mulheres e 20s para homens para o Tempo Máximo de Fonação (TMF). Nesse momento, foi possível realizar a análise perceptivoauditiva da voz e anotar a presença/ausência de rugosidade durante a emissão da vogal. Solicitou-se também a emissão de uma tosse espontânea forte para avaliar a eficiência subjetiva na produção da tosse sob comando (eficiente/fraca) para uma eventual limpeza faríngea. Todas as alterações foram descritas e anotadas para dar prosseguimento a avaliação instrumental da deglutição.

#### Avaliação instrumental da deglutição

O exame da videoendoscopia da deglutição foi realizado por um médico residente, acompanhado por um otorrinolaringologista responsável e pelo fonoaudiólogo com experiência em disfagia orofaríngea, em consonância com o protocolo da instituição. Foi utilizado um nasofibroscópio flexível, marca Olympus^®^ de 3,2mm de diâmetro, modelo LF-P com microcâmera e fonte de luz acoplados. O paciente foi orientado a permanecer na posição sentada durante toda realização do exame, não sendo utilizado anestésico tópico durante a introdução do instrumento na cavidade nasal até a região de hipofaringe. A sensibilidade faríngea foi verificada com o toque do nasofibroscópio em região de epiglote e observada reação de constrição faríngea. Após as condutas de análise das estruturas, realizadas pelo médico, o fonoaudiólogo ofertava alimentos, saborizados artificialmente com suco dietético em pó, corados artificialmente com anilina azul e espessados por meio de um produto instantâneo a base de amido de milho, além de, ao final, ser oferecida uma porção de biscoito salgado de 8g por demanda livre.

As consistências alimentares avaliadas de acordo com a classificação *International Dysphagia Diet Standartisation Initiative* (IDDSI)^(13)^ seguiram a ordem: 2 (líquido levemente espessado), 4 (líquido extremamente espessado) e 0 (líquido ralo) em três ofertas em uma colher metálica de 5mL, enquanto o nível 7 (sólido regular) foi uma oferta de porção única.

Os três profissionais anteriormente citados, com experiência na realização do exame, foram responsáveis por interpretar, avaliar simultaneamente, por consenso e concluíram se havia presença de deglutições múltiplas, escape oral posterior, resíduos faríngeos em região de valéculas e/ou seios piriformes, de acordo com a escala *Yale Pharyngeal Residue Severity Rating Scale* (YPRSRS)^(14)^ (1 - Nenhum, 2 - Vestígio faríngeo, 3 - Resíduo leve, 4 - Resíduo moderado, 5 - Resíduo grave). Também foi avaliada a presença de penetração laríngea e aspiração laringotraqueal. Foram considerados para a análise, a partir da primeira oferta, os seguintes parâmetros: deglutições múltiplas, considerada a partir de mais de duas tentativas de deglutir a mesma oferta^(15)^; escape oral posterior, pela presença do escape prematuro do alimento em região de hipofaringe antes de desencadear a reação de deglutição^(15)^; resíduo faríngeo, por meio da identificação de presença residual de alimento corado em região de valéculas e/ou seios piriformes, após a deglutição da primeira oferta em diante^(14)^; penetração laríngea, via observação de presença residual de alimento corado em região de pregas vocais^(16)^; e aspiração laringotraqueal, quando houvesse resíduo de alimento corado abaixo das pregas vocais^(16)^. Toda análise aconteceu em tempo real e as imagens foram armazenadas em um computador do próprio ambulatório, para serem revisadas quantas vezes os profissionais julgassem necessárias após a realização do exame.

O nível de ingestão oral foi avaliado pelos profissionais após a realização do exame, utilizando-se a escala *Functional Oral Intake Scale* (FOIS)^(17)^ baseado na análise do exame, existência e necessidade de espessamento de líquidos. As pontuações na escala FOIS variam de 1 (nada por via oral) a 7 (ingestão total por via oral sem restrições).

#### Risco nutricional

Para avaliar o risco nutricional, uma nutricionista do serviço foi responsável por aplicar o instrumento *Malnutrition Screening Tool* (MST)^(18)^, traduzido e adaptado para o português, constituído por três perguntas com relação a autopercepção de perda de peso e perda de apetite para se alimentar no último mês. O MST é um instrumento acessível e rápido de se aplicar em adultos na admissão hospitalar, em que, valores iguais ou superiores a dois representam risco nutricional e necessidade de avaliação nutricional mais detalhada.

### Análise dos dados

Para análise dos dados, foi utilizada a estatística descritiva e inferencial por meio de medidas de tendência central, proporções e frequências. Foi utilizado o teste Kolmogorov-Smirnov para verificar a distribuição normal nas variáveis quantitativas, seguidos do teste de Mann-Whitney na comparação dos protocolos utilizados e na análise dos números de dentes pré e pós uso da reabilitação oral. Para as variáveis qualitativas, como “avaliação fonoaudiológica” e “sinais faríngeos videoendoscópios”, aplicou-se o teste Qui-quadrado de Pearson ou teste Exato de Fisher, a depender da frequência esperada por cada célula ser superior ou igual a 5. Para todos os testes considerou-se o nível de significância de 0,05.

## RESULTADOS

Todos os participantes do grupo de idosos sem o diagnóstico apresentavam disfagia por causa idiopática em processo de investigação. Dos 22 indivíduos disfágicos do GDP, 18,1% estavam no estágio I, 54,5% no estágio II e 31,8% no estágio III, de acordo com a escala Hoehn e Yahr^(8)^. Na Tabela 1 está apresentada a relação dos achados da avaliação fonoaudiológica entre os grupos, em que os idosos com Parkinson apresentaram controle postural instável, força de língua reduzida, TMF reduzido e tosse espontânea fraca.

**Tabela 1:** Relação entre idade, gênero e achados da avaliação fonoaudiológica entre os grupos

Variáveis	Grupos		Valor - p
	com Parkinson	sem Parkinson	
	n = 22 (%)	n = 32 (%)	
Idade (anos)	71,32 (± 8,5)	70,50 (± 7,8)	
Gênero			
Masculino	14 (63,6)	5 (15,6)	
Feminino	8 (36,4)	27 (84,4)	
**Avaliação fonoaudiológica**			
Controle postural (sentado)			
Adequado	13 (59,1)	29 (90,6)	0,009**
Instável	9 (40,9)	3 (9,4)	
Mobilidade de língua			
Adequado	16 (72,7)	27 (84,4)	0,324
Reduzido	6 (27,3)	5 (15,6)	
Força de língua			
Adequado	10 (45,5)	27 (84,4)	0,002^*^
Reduzido	12 (54,5)	5 (15,6)	
Tempo Máximo de Fonação			
Adequado	7 (31,8)	25 (78,1)	0,001*
Reduzido	15 (68,2)	7 (21,9)	
Rugosidade			
Ausente	12 (54,5)	20 (62,5)	0,559
Presente	10 (45,5)	12 (37,5)	
Tosse espontânea			
Eficiente	16 (72,7)	30 (93,8)	0,041^**^
Fraca	6 (27,3)	2 (6,3)	

Todos os dados são apresentados em número (%) ou média (desvio padrão).

* Qui-quadrado de Pearson;

** Teste Exato de Fisher

**Legenda:** n(%)= frequência absoluta e relativa

A relação do estado oral, descrito na Tabela 2, apresentou que 54.5% dos indivíduos com Parkinson não utilizavam nenhum tipo de prótese dentária, enquanto que no grupo de idosos sem o diagnóstico, 53.1% não utilizavam. O número de dentes pré e pós uso de prótese dentária foi menor no grupo com Parkinson, além do número de dentes após uso de prótese tender a ser maior para ambos os grupos.

**Tabela 2:** Relação do estado oral entre os grupos

Estado oral	Grupos		**Valor - p**
	com Parkinson	sem Parkinson	
	n = 22 (%)	n = 32 (%)	
Número de dentes sem próteses	13 (8-20,2)	18 (12-24)	0,047^*^
Número de dentes com próteses	20 (9,7-24,2)	24 (20,5-26)	0,015*

Índice de Eichner				
Classe A	12 (54,5)	22 (68,8)	0,288	
Classe B ou C	10 (45,5)	10 (31,3)		
Estase salivar				
Ausente	21 (95,5)	32 (100)	0,407	
Presente	1 (4,5)	0 (0,0)		

Todos os dados são apresentados em mediana (intervalo interquartil 25-75) ou número (%)

* Teste de Mann-Whitney

**Legenda:** n(%)= Frequência absoluta e relativa

Na relação dos achados da videoendoscopia da deglutição e de risco nutricional, apresentada na Tabela 3, verificou-se redução na sensibilidade faríngea ao toque em 40,9% dos participantes com DP. Na consistência de líquido ralo (nível 0) houve maior ocorrência de resíduos faríngeos, penetração laríngea e aspiração laringotraqueal. No líquido levemente espessado (nível 2) houve maior ocorrência de resíduos faríngeos e penetração laríngea. Porém, não houve diferença entre os sinais faríngeos de disfagia nas consistências de líquido extremamente espessado (nível 4) e sólido (nível 7) entre os grupos. Observa-se também que os indivíduos do GDP foram os únicos que apresentaram ocorrências de penetração e aspiração em todas as consistências alimentares avaliadas.

**Tabela 3:** Relação entre os sinais faríngeos da videoendoscopia da deglutição, nível de ingestão oral e o risco nutricional entre os grupos

Sinais faríngeos por nível de consistência alimentar (IDDSI)	Grupos		Valor - p
	com Parkinson	sem Parkinson	
	n = 22 (%)	n = 32 (%)	
Sensibilidade faríngea ao toque			
Preservada	13 (59,1)	28 (87,5)	0,016*
Reduzida	9 (40,9)	4 (12,5)	
Líquido ralo (nível 0)			
Deglutições múltiplas			
Sim	1 (4,5)	1 (3,1)	0,653
Não	21 (95,5)	31 (96,9)	
Escape oral posterior			
Sim	11 (50,0)	11 (34,4)	0,251
Não	11 (50,0)	21 (65,6)	
Resíduos faríngeos			
Sim	14 (63,3)	11 (34,4)	0,034*
Não	8 (36,4)	21 (65,6)	
Penetração laríngea			
Sim	7 (31,8)	0 (0,0)	0,001**
Não	15 (68,2)	32 (100)	
Aspiração laringotraqueal			
Sim	5 (22,7)	0 (0,0)	0,008^**^
Não	17 (77,3)	32 (100)	
Líquido levemente espessado (nível 2)			
Deglutições múltiplas			
Sim	1 (4,5)	1 (3,1)	0,653
Não	21 (95,5)	31 (96,9)	
Escape oral posterior			
Sim	12 (54,5)	13 (40,6)	0,313
Não	10 (45,5)	19 (59,4)	
Resíduos faríngeos			
Sim	17 (77,3)	12 (37,5)	0,004*
Não	5 (22,7)	20 (62,5)	
Penetração laríngea			
Sim	4 (18,2)	0 (0,0)	0,023**
Não	18 (81,8)	32 (100)	
Aspiração laringotraqueal			
Sim	2 (9,1)	0 (0,0)	0,161
Não	20 (90,9)	32 (100)	
Líquido extremamente espessado (nível 4)			
Deglutições múltiplas			
Sim	0 (0,0)	1 (3,1)	0,593
Não	22 (100)	31 (96,9)	
Escape oral posterior			
Sim	14 (63,6)	10 (31,2)	0,019^*^
Não	8 (36,4)	22 (68,8)	
Resíduos faríngeos			
Sim	13 (59,1)	12 (37,5)	0,118
Não	9 (40,9)	20 (62,5)	
Penetração laríngea			
Sim	3 (13,6)	0 (0,0)	0,062
Não	19 (86,4)	32 (100)	
Aspiração laringotraqueal			
Sim	2 (9,1)	0 (0,0)	0,161
Não	20 (90,9)	32 (100)	
Sólido normal (nível 7)			
Deglutições múltiplas			
Sim	1 (4,5)	1 (3,1)	0,653
Não	21 (95,5)	31 (96,9)	
Escape oral posterior			
Sim	4 (18,2)	3 (9,4)	0,425
Não	18 (81,8)	29 (90,6)	
Resíduos faríngeos			
Sim	4 (18,2)	3 (9,4)	0,425
Não	18 (81,8)	29 (90,6)	
Penetração laríngea			
Sim	3 (13,6)	0 (0,0)	0,062
Não	19 (86,4)	32 (100)	
Aspiração laringotraqueal			
Sim	2 (9,1)	0 (0,0)	0,161
Não	20 (90,9)	32 (100)	
Gravidade dos resíduos (YPRSRS)	2.7 (± 1,1)	1.4 (± 0,5)	<0,001***
FOIS	5.1 (± 1,2)	6.0 (± 0,8)	0,006^***^
MST	1.0 (± 1,1)	0.2 (± 0,6)	0,004***

Todos os dados são apresentados em número (%) ou média (desvio padrão)

* Teste do Qui-quadrado;

** Teste Exato de Fisher;

*** Teste Mann-Whitney

**Legenda:** n(%)= Frequência absoluta e relativa; IDDSI = International Dysphagia Diet Standardisation Initiative; YPRSRS = Yale Pharyngeal Residue Severity Rating Scale; FOIS = Functional Oral Intake Scale; MST = Malnutrition Screening Tool

Na análise da gravidade da presença dos resíduos em valéculas e/ou seios piriformes a partir da escala YPRSRS, observou-se diferença na classificação entre os grupos, com presença de resíduos em grau “vestígio a resíduo leve” (YPRSRS 2-3) no grupo com DP. Além disso, notou-se também que o GDP, após o exame instrumental, apresentou FOIS nível 5 (via oral total com múltiplas consistência, porém com necessidade de preparo especial ou compensações) e em risco nutricional (Tabela 3).

## DISCUSSÃO

O mecanismo exato que desencadeia a disfagia na DP ainda não é claro, porém, diversos estudos demonstram que durante a progressão da doença ocorrem diferentes alterações neuromusculares associadas a função de deglutição, que resultam em um mecanismo de regulação da deglutição central e periférica prejudicados^(17)^. Por isso, o presente estudo propôs comparar o estado oral, a função de deglutição por meio da avaliação instrumental, fonoaudiológica e o risco nutricional entre idosos disfágicos com e sem diagnóstico de doença de Parkinson.

Na avaliação fonoaudiológica, encontrou-se diferença significativa na instabilidade do controle postural, força de língua reduzida, TMF reduzido e ineficiência na produção da tosse. Esses achados clínicos corroboram a compreensão de aspectos envolvidos na função de deglutição prejudicados em relação a idosos sem o comprometimento^(9)^.

A força de língua foi uma medida importante que houve diferença significativa entre os grupos, tornando-se um achado consistente com as evidências prévias nessa população. Assim, a redução da força de língua tem sido associada à DP por meio de medidas subjetivas como a avaliação fonoaudiológica e, por medidas objetivas como a pressão isométrica de língua em kilopascal (KPa), que não pode ser modificada com o tratamento medicamentoso com estimulação dopaminérgica^(19,20)^. Além de ser um preditor clínico da eficiência da deglutição utilizado em pesquisas atuais, a baixa pressão entre a língua e o palato também contribuem para as dificuldades em gerenciar e transportar o bolo alimentar em cavidade oral^(21)^. Entende-se também que, a pressão máxima da língua está significativamente diminuída nos estágios mais avançados da doença, além de culminar em uma etapa com aumento de queixas clínicas da deglutição nessa população^(22)^.

O TMF e a eficiência da tosse foram parâmetros clínicos pesquisados por serem confiáveis e conferir ao fonoaudiólogo informações importantes sobre a coaptação das pregas vocais e a eficiência no reflexo de proteção das vias aéreas inferiores. O GDP apresentou diferença entre o TMF e a eficiência da tosse, o que sugere baixa resistência na coaptação de pregas vocais e um reflexo de tosse fraco para ejetar possíveis materiais aspirados na laringe. Esses resultados demonstram que o grupo com DP apresenta menor proteção e segurança na deglutição em comparação àqueles sem o diagnóstico, com maiores riscos de eventuais aspirações silenciosas ao ingerir fluidos, devido o reflexo de tosse fraco e a diminuição no mecanismo sensorial em região faríngea evidenciado em outros estudos^(22,23)^.

Outro aspecto que fortalece a perspectiva de uma diminuição sensorial em fase faríngea no presente estudo, foi a diferença encontrada na sensibilidade faríngea ao toque do nasofibroscópio durante o exame instrumental da deglutição, em que os pacientes com DP em sua maioria não apresentaram reflexo de constrição. Por serem aspectos importantes relacionados à segurança da deglutição, visto que o paciente necessita de mecanismos sensoriais para desencadear o reflexo de deglutição e ejetar resíduos remanescentes, sua diminuição repercute negativamente na eficiência e coordenação do bolo alimentar em fase faríngea^(24)^. Esse dado também colabora para uma compreensão mais profunda de como a fase faríngea, determinada por mecanismos involuntários, pode estar prejudicada tanto na sua aferência quanto na sua eferência na fisiopatologia da DP. Embora acreditasse que a disfagia na DP emergia exclusivamente de uma musculatura debilitada, com movimentos ineficientes em fase preparatória oral e oral propriamente dita, muitos estudos dedicam-se a descrever a fase faríngea da deglutição relativamente preservada nos estágios iniciais da disfagia, porém com uma anormalidade progressiva na integração sensório-motora entre fase oral e faríngea relevante para o quadro disfágico da doença^(25)^.

Com relação ao estado oral, o número de dentes pré e pós uso de reabilitação oral foi diferente nos idosos com o diagnóstico em comparação àqueles sem. O estado oral, apesar de pouco pesquisado em amostras grandes na DP, há evidências de um enfraquecimento à medida que a doença progride, com presença de doenças periodontais e cáries dentárias, o que resulta em dentes mais frágeis e ausentes na cavidade oral^(26)^. Com a saúde bucal comprometida e a função motora oral prejudica, o indivíduo disfágico com DP terá maiores complicações na ingestão oral com declínio no quadro disfágico^(27)^. Por haver menos dentes e, por consequência, menos contatos oclusais, o paciente com DP necessitará de maior tempo de mastigação e processamento do alimento, ou até mesmo mudanças no aporte calórico por preferir consistências alimentares mais fáceis de ingerir^(27)^.

Apesar da análise do contato oclusal entre molares não ter sido diferente entre os grupos, essas informações fornecem uma caracterização de que os indivíduos disfágicos com DP nesta amostra apresentaram ausências dentárias, porém, essas ausências foram compensadas com o uso de reabilitação oral no momento de avaliação do IE, principalmente em região de molares, responsável por fornecer apoio oclusal e triturar os alimentos. Nesse contexto, o uso de próteses dentárias contribui para melhorias tanto na fase preparatória oral, quanto para a fase oral propriamente dita da deglutição. Pois, há relatos de que a ausência de próteses em idosos edêntulos alterou a estrutura anatômica e os movimentos funcionais em cavidade oral, o que resultou em um transporte do bolo alimentar alterado^(28)^.

A análise dos sinais faríngeos da disfagia foi realizada a partir do exame da VED nas diferentes consistências alimentares. Quando comparado entre os grupos, houve diferença nas consistências de líquido ralo e líquido levemente espessado, na qual, o grupo com DP apresentou resíduos faríngeos, penetração laríngea e aspiração laringotraqueal. Esses resultados demonstraram que as consistências líquidas, ou seja, aquelas mais fáceis de fluir, apresentaram riscos significativos para a segurança da deglutição na DP, em comparação ao grupo de idosos disfágicos, que não apresentaram ocorrências de penetração ou aspiração^(23)^. Assim, os dados sugerem que o líquido extremamente espessado pode ser uma alternativa para aumentar a segurança na deglutição, além de contribuir com a compreensão de que os idosos com Parkinson, mesmo no estágio inicial e intermediário (I, II e III) da doença, possuem risco de aspirar líquidos.

O grupo com DP apresentou, em sua maioria, resíduos em grau “vestígio a leve” (2-3), que corrobora a outras evidências de que a disfagia na DP está intrinsecamente relacionada com a presença de resíduos após a deglutição, que poderia ser explicada pela redução na retropropulsão de língua e no atraso do reflexo de deglutição^(24)^. A mensuração dos resíduos na DP ainda não é consenso entre os estudos, com heterogeneidade no método de avaliação aplicado ou na amostra recrutada, porém, é fato que a mudança na consistência alimentar diminui sua ocorrência^(23,29)^.

No presente estudo, houve diferença no nível de ingestão oral e no risco nutricional, o que sugere que os idosos com DP apresentam restrições na ingestão oral e perda de peso relevante. A perda de peso e a desnutrição estão diretamente relacionados ao balanço energético negativo, ou seja, uma ingestão menor que o gasto de energia, que no caso da disfagia, ocorre devido às modificações e restrições nas escolhas dos alimentos pela dificuldade em engolir. Portanto, há evidências que a população com DP possuem maiores riscos de desnutrição em comparação a idosos disfágicos, pelo declínio progressivo da eficiência e segurança da função de deglutição e pelo estado oral prejudicado^(30)^. Apesar da amostra ser constituída por idosos, a desnutrição é considerada um fator secundário e posterior às manifestações disfágicas e que podem ser monitoradas constantemente como marcadores do quadro disfágico nessa população^(30)^. A prevalência da desnutrição na DP ainda é heterogênea de acordo com o método utilizado, seja ele com medidas antropométricas, bioquímicas ou por instrumentos de rastreio^(30)^.

Como limitação do estudo podemos citar o tamanho da amostra reduzido para ambos os grupos, a falta de dados sobre o tipo e localização das próteses dentárias utilizadas e a avaliação nutricional não conter dados antropométricos e/ou bioquímicos para comparação, visto que essas medidas poderiam aumentar o número de indivíduos com risco nutricional. Importante também mencionar o número desigual de participantes nos diferentes estágios da DP, o tempo do diagnóstico e outras informações sobre os medicamentos utilizados, como a investigação se os participantes estavam ou não sob efeito dos medicamentos durante a realização do exame. Como pontos fortes, podemos citar que os resultados do estudo são capazes de fornecer informações relevantes sobre a diferença nos achados da avaliação fonoaudiológica e instrumental da deglutição entre idosos disfágicos com e sem o diagnóstico, de modo que seja possível levantar novas hipóteses de pesquisa clínica entre os grupos.

## CONCLUSÃO

Houve diferença entre os grupos entre o estado oral com relação ao número de dentes, na avaliação fonoaudiológica com força de língua reduzida, tempo máximo de fonação reduzido, tosse espontânea fraca e o nível de ingestão oral, além de sinais faríngeos de resíduos, penetração laríngea e aspiração laringotraqueal nas consistências de líquido ralo e líquido levemente espessado. O grupo com idosos disfágicos com doença de Parkinson apresentaram, em sua maioria, risco nutricional.
